# Perceived and Objective Measures of Neighborhood Walkability and Physical Activity among Adults in Japan: A Multilevel Analysis of a Nationally Representative Sample

**DOI:** 10.3390/ijerph121013350

**Published:** 2015-10-23

**Authors:** Tomoya Hanibuchi, Tomoki Nakaya, Mayuko Yonejima, Kaori Honjo

**Affiliations:** 1 School of International Liberal Studies, Chukyo University, 101 Tokodachi, Kaizu-Cho, Toyota, Aichi 470-0393, Japan; 2 Department of Geography and Research Institute for Disaster Mitigation of Urban Cultural Heritage, Ritsumeikan University, 58 Komatsubara Kitamachi, Kita-Ku, Kyoto 603-8341, Japan; E-Mail: nakaya@lt.ritsumei.ac.jp; 3 Kinugasa Research Organization, Ritsumeikan University, 56-1 Toji-in Kitamachi, Kita-Ku, Kyoto 603-8577, Japan; E-Mail: teddy.mayuko@gmail.com; 4 Global Collaboration Center, Osaka University, 2-7 Yamadaoka, Suita, Osaka 565-0871, Japan; E-Mail: khonjo@glocol.osaka-u.ac.jp

**Keywords:** neighborhoods, physical activity, geographic information systems, multilevel analysis, Japan

## Abstract

Although associations between a person’s neighborhood and their health have been studied internationally, most studies have been limited to a few cities or towns. Therefore, we used a nationally representative sample to explore whether perceived and objective neighborhood walkability was associated with the physical activity of residents. Data were analyzed from the Japanese General Social Surveys of 2010 (*n* = 2395; 1114 men and 1281 women). Perceived walkability was scored using factor analysis for the respondents’ perceptions of neighborhood conditions, while objective walkability was measured using the geographic information system approach. Finally, multilevel logistic regression analysis was performed to examine whether neighborhood walkability was associated with the frequency of leisure-time physical activity (LTPA) among respondents. We found that perceived walkability was positively associated with the frequency of LTPA (odds ratio of the highest quartile was 1.53 (1.14–2.05) compared with the lowest quartile); however, objective walkability showed no association. When stratified by gender, an association between perceived walkability and LTPA was observed among women, but only a marginally significant association was present between objective walkability and LTPA among men. We conclude that the association between neighborhood walkability and LTPA can be partially generalized across Japan.

## 1. Introduction

The health benefits of physical activity (PA) have been well documented, including reduction in mortality risk [1,2] and prevention of chronic diseases [3]. Therefore, efforts should be made to increase PA, and this can be aided by examining the factors associated with increased PA. One such factor is the neighborhood in which people live. Indeed, a large number of empirical studies and review articles have been published over recent decades, and these have indicated that there is a beneficial relationship between the neighborhood built environment (BE) and PA [4,5,6,7,8,9]. However, geographical locations can significantly influence the association between BE and PA. Although studies have been implemented worldwide, most were limited to specific global or national areas.

At the global level, much of the available research has been conducted in Western societies, particularly the US [4,10,11]. However, even when these show statistically significant associations between BE and PA, they cannot be assumed to be generalizable to other societies. To improve this generalizability and facilitate cross-national comparison, additional studies are therefore necessary beyond Western countries [12,13], particularly in Asian countries, where few studies have been reported. In Japan, researchers have recently reported on the association between perceived BE and PA [13,14,15,16,17,18], while a few others have objectively analyzed neighborhood environments in relation to PA [19,20,21].

At the national level, most studies have been limited to a few cities or towns. This is particularly the case when objectively measuring the neighborhood BE and its relation to health because of the difficulties of data collection on a nationwide basis [22]. To date, only a few studies have examined these issues, either by examining the geographical distribution of BE in relation to health for small areas [23,24] or by analyzing nationally representative samples linked with neighborhood indicators [24,25,26]. For example, Cummins and Fagg [24] used data from a nationally representative sample in England, together with neighborhood data on green space and deprivation, to assess the association between neighborhood green space and weight status. To enhance generalizability and comparability, a neighborhood study should examine small areas on a nationwide basis.

In this study we aimed to test the hypothesis that there is an association between neighborhood walkability and PA at a national level in Japan. Therefore, we used a nationally representative sample linked with neighborhood indicators across Japan, and we explored whether neighborhood walkability was associated with PA levels. Additionally, some review articles [4,12] indicated that both perceived and objective measures of BE should be included, while a recent study in Japan reported on the importance of gender differences in the association between neighborhood BE and PA [18,19]. Therefore, analyses were also performed for both perceived and objective indicators of walkability, and the data were stratified by gender.

## 2. Methods

### 2.1. Study Design

We performed a secondary analysis using data from the Japanese General Social Surveys (JGSS) for 2010. This cross-sectional social survey is used to study the attitudes and behaviors of the Japanese population. The survey population included individuals aged between 20 and 89 years living in Japan. A nationally representative sample was selected from among this population using a two-stage stratified random sampling design. 

An ethical review was not required because the JGSS data are available in the public domain, and researchers can apply for access via the Social Science Japan Data Archive. Secondary analysis for academic purposes was permitted because the archive provides individual respondent data in a manner that individual respondents cannot be identified. More details on the JGSS are available at their website [27]. 

### 2.2. Data

The two-stage stratified random sampling was conducted as follows. In the first stage, 600 survey locations (geographical areas defined by census divisions) were randomly sampled from across Japan, according to the strata stratified by regional block and population size. At the second stage, approximately 15 individuals were systematically selected from each survey location using the Basic Resident Registers. Then, data were collected through a combination of interviews and self-administered questionnaires. Two self-administered questionnaire forms (Forms A and B) were randomly assigned so that half of the individuals received Form A, while the other half received Form B. We used the data of Form B, which contained questions on both residential neighborhoods and health conditions. The number of valid responses was 2496 (official response rate = 62.1%). We then excluded samples with missing data for outcomes (frequency of LTPA), education level, marital status, and perceived or objective neighborhood conditions. The final sample consisted of 2395 subjects (1114 men and 1281 women).

For objective measures of area characteristics, small area codes (*chocho-aza*) were added to the JGSS data to provide details of the survey locations. We applied to the JGSS Research Center for permission to refer to these small area codes. Permission was granted on the understanding that data processing and analysis only be conducted within a building of the JGSS Research Center and that the results of the final analysis be published in a way that the respondents could not be identified.

### 2.3. Outcomes

The frequency of exercise or sport, including walking, was used as the outcome variable and termed LTPA. Respondents were asked “Do you regularly do any exercises or play any sports (walking, swimming, baseball, *etc*.)?” with possible choices of “several times a week, about once a week, about once a month, several times a year, and scarcely any exercise.” LTPA was defined as “1” if the respondents exercised or played sports several times a week and as “0” if they provided any other response.

### 2.4. Perceived Walkability

Although a questionnaire of JGSS-2010 did not include a specific established scale to measure the perceived neighborhood environment, we utilized the below mentioned 10 items. First, respondents were asked “How severe are the following issues in the area of your local residence?” and were provided with a list of four negative factors that included air pollution, water pollution, noise pollution, and obscured sunlight. Respondents were asked to rate each item on a four-point scale from “very severe” to “not severe at all.” Next, on a five-point scale (from “strongly disagree” to “strongly agree”), they were asked the extent to which they agreed or disagreed with each of the following six statements.

- The neighborhood is suitable for exercise such as jogging or walking.
- A large selection of fresh fruits and/or vegetables is available in my neighborhood.
- The neighborhood has adequate public facilities (community center, library, park, *etc*.).
- The neighborhood is safe.
- The neighbors are mutually concerned for each other.
- The neighbors are willing to provide assistance when I am in need.

We then performed exploratory factor analysis with principal axis extraction and varimax rotation to explore all 10 variables. From these, we extracted factors with eigenvalues greater than one, which were considered to represent important dimensions of respondents’ perceptions, including perceived walkability, and the factor scores were used in the subsequent regression analysis as individual level independent variables.

### 2.5. Objective Walkability

In this study, we defined the objective unit of the neighborhood as *chocho-aza* (defined by the 2010 population census of Japan), which is the smallest administrative unit and is roughly comparable to a U.S. census-block group. All our data was based on the National Land Numerical Information (NLNI) and the population census of Japan, as of 2010, unless otherwise stated. Referring to the previous study on the walkability index in Japan [28] and considering the nationwide availability of spatial data, we constructed an objective walkability index for approximately 200,000 neighborhoods across Japan. This index consisted of population density, road density, access to parks, and access to retail areas. For road density, as a proxy for street connectivity, data were taken from the NLNI, which provides data of road density for each tertiary mesh (road length per 1 km^2^). The road density of the neighborhoods was defined by referencing the road density of the tertiary mesh located at the geometric center of each neighborhood. Access to parks and retail areas was measured as the linear distance to the nearest places from the geometric center of each neighborhood. Data on parks were also obtained from the NLNI. We used retail area data for 2011, as originally developed by Akiyama *et al*. [29] (released by Zenrin Co. Ltd., Kitakyushu, Fukuoka, Japan). Clusters of retail stores were based on the point data from the Yellow Pages, with retail areas defined as polygons. Each of the four indexes was divided into decile groups from one (least walkable) to 10 (most walkable) and added to each other. Finally, scores ranging from four to 40 were linked with individual samples.

### 2.6. Covariates

Individual attributes, including age (20–29, 30–39, 40–49, 50–59, 60–69, 70–79, 80–89), education (junior high school, high school, junior/technical college, university or above), marital status (currently married, divorced/widowed, never married), and work status (not working, working) were used as possible confounders for the association between neighborhood walkability and PA. In the statistical models, we also considered “perceived neighborhood pollution” and “perceived neighborhood sociability” to adjust for neighborhood perceptions other than walkability. These variables were also extracted by factor analysis to the 10 variables in Section 2.4 (Perceived Walkability) regarding neighborhood conditions.

For the neighborhood level (*i.e*., *chocho-aza*) covariates, we used a census-based objective index of neighborhood deprivation in Japan [30]. This composite indicator consisted of weighted sums of several poverty-related census variables (as of 2010), such as proportion of elderly couples in households, proportion of elderly single-occupier households, proportion of single-mother households, proportion of rented houses, proportion of sales and service workers, proportion of agricultural workers, proportion of blue-collar workers, and the unemployment rate. Details of this area deprivation index are described elsewhere [30].

### 2.7. Statistical Analyses

The statistical analysis was performed using STATA 12. For bivariate associations between individual/neighborhood variables and the frequency of LTPA, we calculated the percentages of those engaged in LTPA and performed analyses using the chi-square test. We estimated the adjusted odds ratios (ORs) and 95% confidence intervals (CIs) for perceived and objective walkability by multilevel, random intercept, logistic regression models (xtlogit in STATA), using *chocho-aza* as the group variable. Objective neighborhood indices for walkability and deprivation were included as second-level variables in the multilevel models. All variables of perceived and objective neighborhood environment were grouped by quartile and included simultaneously in the models. Analyses were performed for all samples, and were also stratified by gender.

## 3. Results

Factor analysis for the perceptions of the neighborhood yielded three factors with eigenvalues greater than one. The rotated factor loadings are presented in Table 1. The three extracted factors were “perceived neighborhood pollution,” “perceived neighborhood walkability,” and “perceived neighborhood sociability.” These factors were used for the subsequent regression analysis as individual-level independent variables.

**Table 1:** Rotated Factor Loadings (varimax rotation).

Variables	Factor 1	Factor 2	Factor 3
	Pollution	Walkability	Sociability
Low air pollution	0.843	0.069	0.046
Low water pollution	0.846	0.105	0.013
Low noise pollution	0.752	0.113	0.121
Low invasion of access to sunlight	0.677	0.130	0.089
Suitable for exercise	0.128	0.593	0.141
Availability of fresh fruits and/or vegetables	0.102	0.588	0.170
Adequate public facilities	−0.007	0.628	0.045
Safe	0.203	0.687	0.257
Mutually concerned for each other	0.082	0.283	0.842
Willing to provide assistance	0.102	0.203	0.840

**Figure 1:**
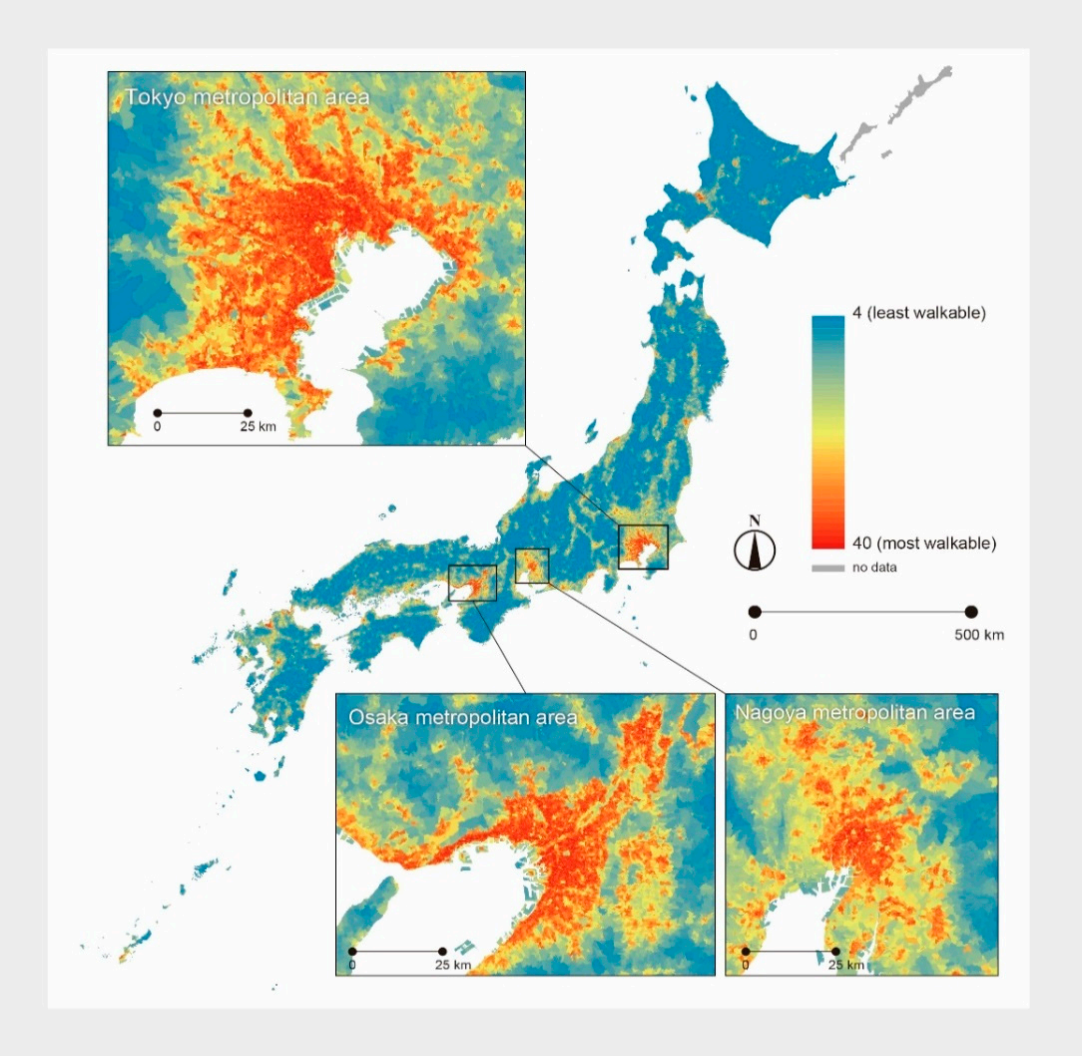
Geographical distribution of objectively measured neighborhood walkability.

Figure 1 shows the geographical distribution of the objective walkability index. As might be expected from the index components, the map indicates that there are clear urban-rural differences; in Japan, the urban areas were more walkable than the rural areas. It should also be noted that the gap in the walkability index was larger between urban and rural areas than between city centers and suburbs within urban areas.

The characteristics of the respondents of JGSS-2010 are presented in Table 2. In total, 26.8% of men and 22.6% of women were engaged in LTPA. Bivariate analysis indicated that individual attributes, including gender, age, marital status, and work status, were related to LTPA; however, although relevant among women, education was not significantly related to LTPA among men. In both genders, the percentages of those engaged in LTPA were higher among the respondents who perceived their neighborhoods as walkable. Objective neighborhood walkability and neighborhood deprivation were not significantly related to LTPA in the bivariate analysis.

**Table 2:** Characteristics of Respondents.

Variables	All		Men		Women	
	*n*	% Having LTPA	*n*	% Having LTPA	*n*	% Having LTPA
**Gender**						
Men	1114	26.8				
Women	1281	22.6				
		*p* < 0.05 **^a^**				
**Age (years)**						
20–29	232	16.4	104	24.0	128	10.2
30–39	377	12.7	170	12.9	207	12.6
40–49	393	15.5	166	16.9	227	14.5
50–59	410	21.5	190	21.1	220	21.8
60–69	518	37.1	253	35.6	265	38.5
70–79	339	38.3	172	42.4	167	34.1
80–89	126	24.6	59	33.9	67	16.4
		*p* < 0.001 **^a^**		*p* < 0.001 **^a^**		*p* < 0.001 **^a^**
**Education**						
Junior high school	366	26.0	190	24.2	176	27.8
High school	1115	25.0	462	26.4	653	24.0
Junior/technical college	364	17.3	108	22.2	256	15.2
University or above	550	27.5	354	29.9	196	23.0
		*p* < 0.01 **^a^**		n.s. **^a^**		*p* < 0.01 **^a^**
**Marital status**						
Currently married	1733	25.8	828	28.5	905	23.3
Divorced/Widowed	283	26.5	77	26.0	206	26.7
Never married	379	17.4	209	20.1	170	14.1
		*p* < 0.01 **^a^**		*p* < 0.05 **^a^**		*p* < 0.01 **^a^**
**Work status**						
Not working	935	34.8	339	44.8	596	29.0
Working	1460	18.0	775	18.8	685	17.1
		*p* < 0.001 **^a^**		*p* < 0.001 **^a^**		*p* < 0.001 **^a^**
**Perceived neighborhood pollution**						
Q1 (highest pollution)	598	22.1	256	21.5	342	22.5
Q2	594	24.4	264	26.9	330	22.4
Q3	606	24.4	293	29.0	313	20.1
Q4 (lowest pollution)	597	27.3	301	28.9	296	25.7
		n.s. **^a^**		n.s. **^a^**		n.s. **^a^**
**Perceived neighborhood walkability**						
Q1 (least walkable)	600	19.3	281	22.8	319	16.3
Q2	597	21.1	278	19.8	319	22.3
Q3	595	27.4	278	30.9	317	24.3
Q4 (most walkable)	603	30.3	277	33.6	326	27.6
		*p* < 0.001 **^a^**		*p* < 0.001 **^a^**		*p* < 0.01 **^a^**
**Perceived neighborhood sociability**						
Q1 (least sociable)	603	20.9	278	25.2	325	17.2
Q2	601	23.1	301	22.9	300	23.3
Q3	605	28.1	302	31.8	303	24.4
Q4 (most sociable)	586	26.1	233	27.0	353	25.5
		*p* < 0.05 **^a^**		*p* < 0.1 **^a^**		*p* < 0.1 **^a^**
**Objective neighborhood walkability**						
Q1 (least walkable)	706	22.7	354	22.6	352	22.7
Q2	668	26.0	307	29.3	361	23.3
Q3	542	24.4	233	26.6	309	22.7
Q4 (most walkable)	479	25.5	220	30.0	259	21.6
		n.s. **^a^**		n.s. **^a^**		n.s. **^a^**
**Neighborhood deprivation**						
Q1 (least deprived)	571	25.0	276	27.9	295	22.4
Q2	578	25.4	264	27.3	314	23.9
Q3	612	23.4	278	25.5	334	21.6
Q4 (most deprived)	634	24.4	296	26.4	338	22.8
		n.s. **^a^**		n.s. ^b^		n.s. ^b^

**^a^** *p* value for chi-square test.

The results of multilevel logistic regression analysis are shown in Table 3. Individual attributes, except for marital status, were associated with LTPA. The following groups were more likely to be engaged in LTPA: men; those aged 60–79 years; those who graduated from high school and university; and those not working. Age was not related to LTPA among men, while education was not related to LTPA among women. After adjustment for the covariates and after accounting for the clustering of data, perceived walkability was positively and significantly associated with LTPA. The OR of the perceived walkability was 1.53 (1.14–2.05) for the highest quartile compared with the lowest quartile. In the stratified analysis, this significant association remained among women (1.70, 1.12–2.59), but not among men (1.41, 0.91–2.16). Perceived neighborhood sociability was also related to the increased frequency of LTPA, but the associations were not linear. Although objective walkability was not significantly associated with LTPA, the associations were marginally significant among men, with ORs of 1.43 (0.95–2.16) for Q2, 1.17 (0.74–1.84) for Q3, and 1.48 (0.94–2.35) for Q4. Perceived neighborhood pollution and neighborhood deprivation were not associated with LTPA for either gender.

**Table 3:** Results of Multilevel Logistic Regression Analysis: Adjusted Odds Ratios and 95% Confidence Intervals for Leisure-time Physical Activity.

Variables	All				Men		Women		
	OR	(95% CI)			OR	(95% CI)	OR		(95% CI)
**Gender**									
Men	1.00								
Women	0.77 *	(0.62–0.96)							
**Age (years)**									
20–29	1.00				1.00		1.00		
30–39	0.80	(0.48–1.34)			0.57	(0.28–1.18)	1.38		(0.62–3.05)
40–49	1.06	(0.63–1.78)			0.82	(0.40–1.70)	1.74		(0.78–3.88)
50–59	1.46	(0.88–2.41)			1.00	(0.49–2.03)	2.65 *		(1.20–5.87)
60–69	2.83 ***	(1.71–4.67)			1.67	(0.82–3.42)	5.37 ***		(2.45–11.76)
70–79	2.54 **	(1.47–4.38)			1.58	(0.72–3.48)	4.01 **		(1.72–9.34)
80–89	1.38	(0.71–2.68)			1.06	(0.41–2.73)	1.50		(0.52–4.31)
**Education**									
Junior high school	1.00				1.00		1.00		
High school	1.54 **	(1.14–2.08)			2.07 **	(1.30–3.27)	1.19		(0.77–1.83)
Junior/technical college	1.25	(0.83–1.87)			1.79 **^+^**	(0.93–3.44)	0.91		(0.52–1.59)
University or above	1.89 ***	(1.33–2.69)			2.37 **	(1.44–3.88)	1.55		(0.88–2.75)
**Marital status**									
Currently married	1.00				1.00		1.00		
Divorced/Widowed	0.87	(0.63–1.21)			0.64	(0.35–1.16)	1.06		(0.70–1.61)
Never married	0.98	(0.68–1.42)			0.87	(0.52–1.47)	1.07		(0.59–1.93)
**Work status**									
Not working	1.00				1.00		1.00		
Working	0.51 ***	(0.40–0.64)			0.32 ***	(0.21–0.48)	0.65 **		(0.47–0.89)
**Perceived neighborhood pollution**									
Q1 (highest pollution)	1.00				1.00		1.00		
Q2	1.04	(0.77–1.39)			1.23	(0.78–1.93)	0.95		(0.63–1.41)
Q3	1.11	(0.83–1.48)			1.35	(0.87–2.10)	0.91		(0.60–1.36)
Q4 (lowest pollution)	1.17	(0.87–1.56)			1.34	(0.87–2.09)	1.01		(0.67–1.52)
**Perceived neighborhood walkability**									
Q1 (least walkable)	1.00				1.00		1.00		
Q2	1.03	(0.76–1.39)			0.70	(0.44–1.12)	1.43		(0.93–2.19)
Q3	1.31 **^+^**	(0.97–1.77)			1.18	(0.76–1.83)	1.49 **^+^**		(0.97–2.31)
Q4 (most walkable)	1.53 **	(1.14–2.05)			1.41	(0.91–2.16)	1.70 *		(1.12–2.59)
**Perceived neighborhood sociability**									
Q1 (least sociable)	1.00				1.00		1.00		
Q2	1.24	(0.92–1.66)			1.03	(0.67–1.58)	1.51 **^+^**		(0.99–2.32)
Q3	1.30 **^+^**	(0.97–1.74)			1.56 *	(1.02–2.40)	1.14		(0.74–1.76)
Q4 (most sociable)	1.22	(0.91–1.65)			1.06	(0.67–1.68)	1.35		(0.90–2.03)
**Objective neighborhood walkability**									
Q1 (least walkable)	1.00				1.00		1.00		
Q2	1.18	(0.90–1.55)			1.43 **^+^**	(0.95–2.16)	1.04		(0.71–1.53)
Q3	1.12	(0.84–1.50)			1.17	(0.74–1.84)	1.12		(0.75–1.67)
Q4 (most walkable)	1.24	(0.91–1.69)			1.48 **^+^**	(0.94–2.35)	1.03		(0.66–1.60)
**Neighborhood deprivation**									
Q1 (least deprived)	1.00				1.00		1.00		
Q2	1.07		(0.80–1.44)		1.00	(0.65–1.56)	1.19	(0.79–1.81)	
Q3	0.98		(0.73–1.31)		0.93	(0.60–1.45)	1.05	(0.69–1.58)	
Q4 (most deprived)	1.07		(0.80–1.43)		1.14	(0.74–1.76)	1.06	(0.71–1.60)	
ln (variance of random intercept)	−2.90				−1.58		−2.66		
*p* value for variance of random intercept = 0	0.28				0.15		0.36		
Number of observations	2395				1114		1281		
Number of groups	592				514		532		

*** *p* < 0.001, ** *p* < 0.01, * *p* < 0.05, **^+^** *p* < 0.1.

## 4. Discussion

Many studies have examined the association between neighborhood BE and PA, but most of these studies were region-specific and have lacked the ability to generalize to other locations. For example, a few studies have objectively measured neighborhood BE in relation to PA in Japan [19,20,21], and those that have, tend to have focused on data from limited areas. To our knowledge, no researchers have used a nationally representative sample in Japan to demonstrate the association between PA and neighborhood walkability in both perceived and objective measures. The present study sought to address this gap and found that the associations between neighborhood walkability and LTPA can be partially generalized across Japan. However, caution is warranted because a clear association was only seen for perceived walkability among women.

Several reasons can explain the weak associations that we observed. Notably, the outcome variable was self-reported and defined from a single question. It measured only a limited domain of PA (*i.e.*, LTPA) and did not measure transport-related PA, which has also been more associated with neighborhood BE [7,8]. In addition, the outcome variable did not directly distinguish walking from other types of PA. Previous studies have often indicated that the associations between BE and PA can vary according to the domain and type of PAs [13,17]. For example, Tsunoda *et al.* [17] found a positive link between perceived access to recreational facilities and LTPA except for walking, which they considered was because LTPA except for walking often require a specific location, while walking does not.

Another reason would be that because of differences in data availability, our objective walkability differed to that previously used [31,32] and was slightly different from the Japanese measure [28]. For example, we included access to parks, which was not necessarily included in other walkability indexes. However, this inclusion could conversely have contributed to the association with LTPA because open or green spaces have been found to have a beneficial effect on PA [6]. Moreover, access to retail areas may have indirectly reflected access to urban facilities, resulting in a marginal association with LTPA because these will undoubtedly have included recreational facilities. In any case, future research should employ more specific and consistent variables for both PA and BE.

Regarding gender differences, Chen *et al*. [18] have reported that the association between perceived neighborhood environment and walking time or habitual exercise was observed mainly in middle- and old-aged women. Our results are consistent with this for perceived walkability; among men, it is possible to assume that they spend more time away from their neighborhoods (*i.e*., in the workplace) and perceive their residential neighborhood less accurately than women. Our results on objective walkability were also similar to a previous study in Japan [19], despite age group differences. Hanibuchi *et al*. [19] reported that stronger associations existed between objectively measured BE and sports activity among men. They argued that sport events, which may require specific locations or facilities, were more likely to be preferred by men. Since our objective walkability index may reflect such accessibility to parks or recreational facilities, we would have expected an association with LTPA only among men.

Perceived neighborhood sociability was also related to LTPA, but with a non-linear association. Studies in Japan have indicated that low social capital at an individual level, particularly reduced trust of others in the community, was associated with physical inactivity [33]. In another study, it was reported that the social environment increased the odds of engaging in recreational walking [13]. Importantly, our present results are consistent with this association when using a nationally representative sample. In addition, we found no significant association between LTPA and the deprivation level of an area. This result is consistent with the recently published article by Nakaya *et al.* [30], which reported that the association between neighborhood deprivation and total mortality was not mediated by PA or other health behaviors.

The demographic and socioeconomic factors of individuals were strongly associated with LTPA compared with the results for neighborhood environment. Indeed, LTPA was more likely among middle- to older-aged women, men with higher educational statuses, and those not working. Age and working status may be related to the amount of time available for LTPA, while educational status may reflect an interest in a healthy lifestyle coupled with the economic resources to engage in LTPA. Although we used several variables as covariates (see Section 2.6), these factors could also moderate the association between neighborhood walkability and LTPA; therefore, research is needed on the moderating effects of these variables.

Several limitations should be mentioned. First, the cross-sectional design precludes making a causal association between neighborhood walkability and PA [34]. A longitudinal or quasi-experimental study should be considered to achieve this; however, such a study design would be difficult to apply on a national scale. Second, as mentioned above, PA and perceived neighborhood environment were not validated measurement variables, and the components of the objective neighborhood index were somewhat different from previously established measures. Further studies should use established measures of PA and BE to ensure comparability, but should also consider the different types of PA and BE. Third, the definition of neighborhood in our study was based on an administrative unit, and the distance calculation was based on the Euclidean distance. Using buffer zones or units that reflect available activity spaces and measuring distance by reference to the road network could improve the accuracy of spatial analyses in the future. Finally, although we reported that the associations between BE and PA differed by gender, the moderating effects of gender and other demographic and socioeconomic characteristics should be tested in more detail.

## 5. Conclusions

In conclusion, our analysis of using a nationally representative sample in Japan not only showed that perceived walkability was associated with LTPA but also that there were differences between men and women. Women tended to engage in LTPA if they perceived their neighborhoods as walkable, while men only appeared to engage in LTPA if they resided in objectively walkable neighborhoods. The implication is that health policy may need to consider interventions that target improvements in neighborhood conditions if the levels of PA are to be improved. This might include improving physical access to shops, parks, and recreational facilities as well as making residents feel safe in their neighborhoods. Although such changes could improve the walkability of neighborhoods, more studies are necessary to examine the specific associations between the different types of PA and different elements of BE. The generalizability and comparability of the results can be enhanced by performing this research in nationally representative samples.
